# Deciphering the Catalytic Machinery in 30S Ribosome Assembly GTPase YqeH

**DOI:** 10.1371/journal.pone.0009944

**Published:** 2010-04-01

**Authors:** Baskaran Anand, Parag Surana, Balaji Prakash

**Affiliations:** Department of Biological Sciences and Bioengineering, Indian Institute of Technology, Kanpur, Uttar Pradesh, India; Griffith University, Australia

## Abstract

**Background:**

YqeH, a circularly permuted GTPase (cpGTPase), which is conserved across bacteria and eukaryotes including humans is important for the maturation of small (30S) ribosomal subunit in *Bacillus subtilis*. Recently, we have shown that it binds 30S in a GTP/GDP dependent fashion. However, the catalytic machinery employed to hydrolyze GTP is not recognized for any of the cpGTPases, including YqeH. This is because they possess a hydrophobic substitution in place of a catalytic glutamine (present in Ras-like GTPases). Such GTPases were categorized as HAS-GTPases and were proposed to follow a catalytic mechanism, different from the Ras-like proteins.

**Methodology/Principal Findings:**

MnmE, another HAS-GTPase, but not circularly permuted, utilizes a potassium ion and water mediated interactions to drive GTP hydrolysis. Though the G-domain of MnmE and YqeH share only ∼25% sequence identity, the conservation of characteristic sequence motifs between them prompted us to probe GTP hydrolysis machinery in YqeH, by employing homology modeling in conjunction with biochemical experiments. Here, we show that YqeH too, uses a potassium ion to drive GTP hydrolysis and stabilize the transition state. However, unlike MnmE, it does not dimerize in the transition state, suggesting alternative ways to stabilize switches I and II. Furthermore, we identify a potential catalytic residue in Asp-57, whose recognition, in the absence of structural information, was non-trivial due to the circular permutation in YqeH. Interestingly, when compared with MnmE, helix α2 that presents Asp-57 is relocated towards the N-terminus in YqeH. An analysis of the YqeH homology model, suggests that despite such relocation, Asp-57 may facilitate water mediated catalysis, similarly as the catalytic Glu-282 of MnmE. Indeed, an abolished catalysis by D57I mutant supports this inference.

**Conclusions/Significance:**

An uncommon means to achieve GTP hydrolysis utilizing a K^+^ ion has so far been demonstrated only for MnmE. Here, we show that YqeH also utilizes a similar mechanism. While the catalytic machinery is similar in both, mechanistic differences may arise based on the way they are deployed. It appears that K^+^ driven mechanism emerges as an alternative theme to stabilize the transition state and hydrolyze GTP in a subset of GTPases, such as the HAS-GTPases.

## Introduction

GTP binding proteins or GTPases represent one of the most widely distributed protein families [1]. They regulate diverse cellular processes by cycling between an active GTP-bound and an inactive GDP-bound forms [2]. Circularly permuted GTPases (cpGTPases) represent a subset of these proteins and exhibit a curious permutation of sequence motifs[3], [4]. cpGTPases have been implicated in the process of ribosome assembly[5], [6], [7], [8], [9], [10], [11], [12], [13], [14]. Among these, YqeH which is conserved across bacteria and eukaryotes including humans, is shown to be essential for the maturation of small ribosomal subunit (30S) in *Bacillus subtilis* [10], [11]. An ortholog of YqeH in *Arabidopsis thaliana* was mistakenly considered to be a Nitric Oxide Synthase (AtNOS1) and was later shown to be a functional cpGTPase [15], [16], [17], [18]. In conjunction with its participation in ribosome maturation, recently, we showed that it binds 30S ribosomal subunit in a GTP/GDP dependent manner and that the binding requires adjacent RNA binding domains [19]. Further experiments to probe whether it acts as an RNA chaperone for 30S assembly remained inconclusive [19]. So far, investigations aimed at understanding the ribosome interaction showed that all of the bacterial cpGTPases such as YjeQ, YlqF and YqeH exhibit a GTP/GDP dependent ribosome binding. However, the catalytic machinery employed to hydrolyze GTP is not understood for any of them. Hence, understanding this mechanism would augment elucidating the relation between nucleotide binding/hydrolysis and ribosome binding. Here, we have attempted to decipher the catalytic machinery employed by YqeH.

YqeH (and cpGTPases in general) belongs to an interesting class of GTPases termed HAS-GTPases (Hydrophobic Amino acid Substituted for catalytic glutamine GTPases) that lack the catalytic glutamine conserved in a large set of GTPases, including Ras [20]. Gln-61 mutations in Ras are oncogenic [21], [22] and HAS-GTPases, by virtue of a hydrophobic substitution in place of Gln-61, present a paradox to the GTP hydrolysis mechanism observed in Ras-like classical GTPases [20]. This raises questions on how these proteins may hydrolyze GTP. In an earlier study, based on sequence and structural analysis of these proteins, we proposed few possibilities [20]: The potential catalytic residue may be presented (i) from a different region of the G-domain (*in cis*), (ii) from domain(s) adjacent to the G-domain (*in cis*), or (iii) from an interacting protein (*in trans*). While our attempts to examine these possibilities for YqeH were in progress, it was reported that MnmE, another HAS-GTPase, that participates in tRNA modification exhibits a potassium dependent GTP hydrolysis mechanism and that a part of the switch-I region, termed K-loop, coordinates K^+^ ion and shields it from the solvent [23]. The K-loop also harbors a conserved motif ^249^GTTRD^253^ in switch-I region (Thr-251 corresponds to Thr-35 in Ras that coordinates both Mg^2+^ and γ-phosphate) and coordinates the K^+^ ion through main chain interactions via Thr-245 and Ile-247 [23]. Though YqeH and MnmE share only ∼25% sequence identity in the G-domain, YqeH, in addition to characteristic nucleotide binding motifs G1-G3-G4, exhibits the presence of the conserved ^197^GTTLD^201^ motif - a feature similar to the K-loop in the switch-I of MnmE. This prompted us to investigate whether YqeH too would utilize a K^+^ ion to hydrolyze GTP. Here, we show that YqeH indeed exhibits a potassium dependent GTP hydrolysis. Guided by a homology model based on the transition state structure of MnmE, we attempted to probe the role of potassium in GTP hydrolysis and identified a potential catalytic residue, whose role was verified using site-directed mutagenesis. We conclude that unlike Ras-family GTPases, the GTP hydrolysis mechanism employed by YqeH is similar to that seen in MnmE, albeit with variations in the way catalytic residues are deployed.

## Results

### Potassium Dependent GTPase Activity

**Figure 1:**
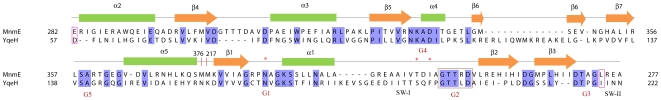
Secondary structure based sequence alignment of the G-domains of MnmE and YqeH. The sequence alignment used to generate the YqeH homology model is shown. Since YqeH is circularly permuted with respect to MnmE, the C-terminal region (282–376 indicated in the figure) of MnmE is relocated to the N-terminus to facilitate alignment with YqeH. α-helices are depicted as green cylinders and β-strands as orange arrows. Sequence motifs G1-G5 are indicated. Conserved residues are highlighted in blue. The catalytic residues in MnmE (E282) and YqeH (D57) are boxed in pink. The hydrophobic substitutions (L274 in MnmE and I219 in YqeH) in place of Q61 of Ras are boxed in pink. Switch-I and II regions are indicated. The conserved motifs in K-loop (GTTRD in MnmE and GTTLD in YqeH) are boxed in brown. Residues that coordinate the potassium ion are indicated by asterisks.

In an earlier analysis, we identified that unlike Ras superfamily GTPases, YqeH lacks the catalytic glutamine in switch-II and was thus categorized as a HAS-GTPase [4]. This prompted us to ask how YqeH hydrolyses GTP in the absence of a catalytic glutamine. Recent structural and biochemical studies on MnmE (earlier termed TrmE), one of the HAS-GTPase members, revealed that it utilizes a potassium ion for GTP hydrolysis [23]. While classical GTPases such as Ras utilize an ‘Arginine finger’ from Ras-GAP to stabilize the transition state, in MnmE, a K^+^ ion fulfills this role, wherein a loop termed the ‘K-loop’ stabilizes the K^+^ ion. Encouraged by the presence of a K-loop like feature in YqeH amidst low sequence identity between the CPG-domain of YqeH and the G-domain of MnmE (∼25% sequence identity), we inquired if YqeH too invokes a potassium dependent GTPase activity (Fig. 1). To test this, GTP hydrolysis by YqeH was measured in the presence of different monovalent salts such as NaCl, KCl, NH_4_Cl, RbCl and CsCl. As shown in Table 1, significant GTP hydrolysis was observed only in presence of KCl, NH_4_Cl and RbCl; while in presence of NaCl there was no activity and that in presence of CsCl was low. The requirement of NH_4_^+^ ions for GTPase activity is also noted for the YqeH ortholog from *Geobacillus sterothermophilus* (GsYqeH) [24]. This suggests that like in MnmE, potassium promotes GTP hydrolysis in YqeH too and raises the possibility that it may act as a GTPase Activating Element (GAE). For classical GTPases like Ras, GAPs (GTPase Activating Proteins) are known to lower the activation energy barrier by stabilizing the transition state [2], [25]. Since the role of GAE is analogous to GAP, the effect of K^+^ ion on transition state stabilization was examined.

**Table 1:** YqeH Activity in Presence of Different Monovalent Cations.

Monovalent Cations (Ionic radius in picometers)	Specific Activity (min^−1^)
Na^+^ (102)	** (0.0001)
K^+^ (138)	1.1844±0.0227 (0.0001)
NH_4_^+^ (147)	0.6437±0.0704 (0)
Rb^+^ (152)	0.5932±0.0156 (0.0001)
Cs^+^ (167)	0.1334±0.0159 (0.0001)

Specific activity is represented as the amount of Pi released for a given concentration of enzyme for a certain time (min). Experiments were conducted in duplicates and were reproduced at least twice. The errors represent the standard deviation from the average. Absorbance was corrected for the background intrinsic GTP hydrolysis and the background GTP hydrolysis (represented as amount of Pi released to the amount of GTP added, per min) is indicated in brackets. ** indicates that the activity could not be measured.

**Figure 2:**
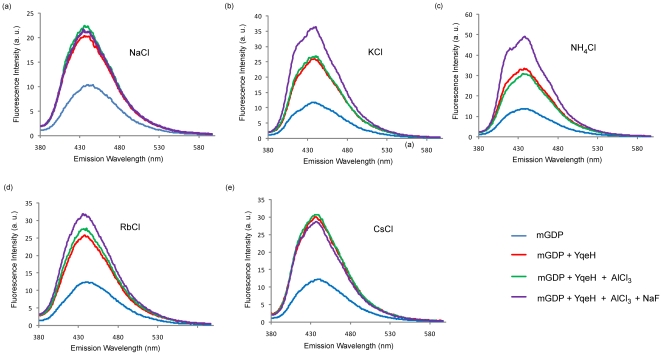
Formation of mGDP·AlF_x_ complex in the presence of various monovalent salts. The influence of (a) NaCl, (b) KCl, (c) NH_4_Cl, (d) RbCl and (e) CsCl on nucleotide binding was examined by measuring fluorescence intensity (in arbitrary units) of mant-GDP (λ_ex_ - 355 nm, λ_em_ - 440 nm). The intrinsic fluorescence by mGDP (blue curve) is enhanced after the addition of YqeH (red curve). This is followed by addition of AlCl_3_ in the same reaction mixture which shows no appreciable change (green). Addition of NaF (purple), however, results in an increased fluroscence in presence of (a) K^+^ (c) NH_4_^+^ and (d) Rb^+^ suggesting the formation of mGDP·AlF_x_ complex in presence of these ions.

Sensitive fluorescent probes like mant-nucleotides are used to study nucleotide binding in GTPases[26]. In several GTPases, GDP·AlF_x_ was shown to mimic the transition state of GTP hydrolysis[22], [26]. Hence, to examine the role of K^+^ ion in transition state stabilization, the proteins were incubated with mant-GDP (mGDP) and AlF_x_, in the presence of K^+^. It is known that enzymes exhibit a tight binding to substrates, in the transition state. In the nucleotide binding experiments, we anticipated that K^+^ ion promoted binding of mGDP·AlF_x_ to YqeH, would be reflected by an increased fluorescence (of mGDP·AlF_x_ complex). Therefore, the fluorescence experiments were conducted in presence of Na^+^, K^+^, NH_4_^+^, Rb^+^ and Cs^+^ ions (Fig. 2). In all conditions, addition of YqeH resulted in enhanced intrinsic fluorescence of mGDP suggestive of nucleotide binding (see blue and red curves in Fig. 2). Following this, addition of AlCl_3_ did not alter this binding (see green curves in Fig. 2). However, further addition of NaF that would promote the formation of AlF_x_, resulted in enhanced fluorescence only in presence of K^+^, NH_4_^+^ and Rb^+^ ions, but not Na^+^ and Cs^+^. This suggests the formation of a transition state analogue GDP·AlF_x_ in the presence of K^+^, NH_4_^+^ and Rb^+^ (see purple curves in Fig. 2).

**Figure 3:**
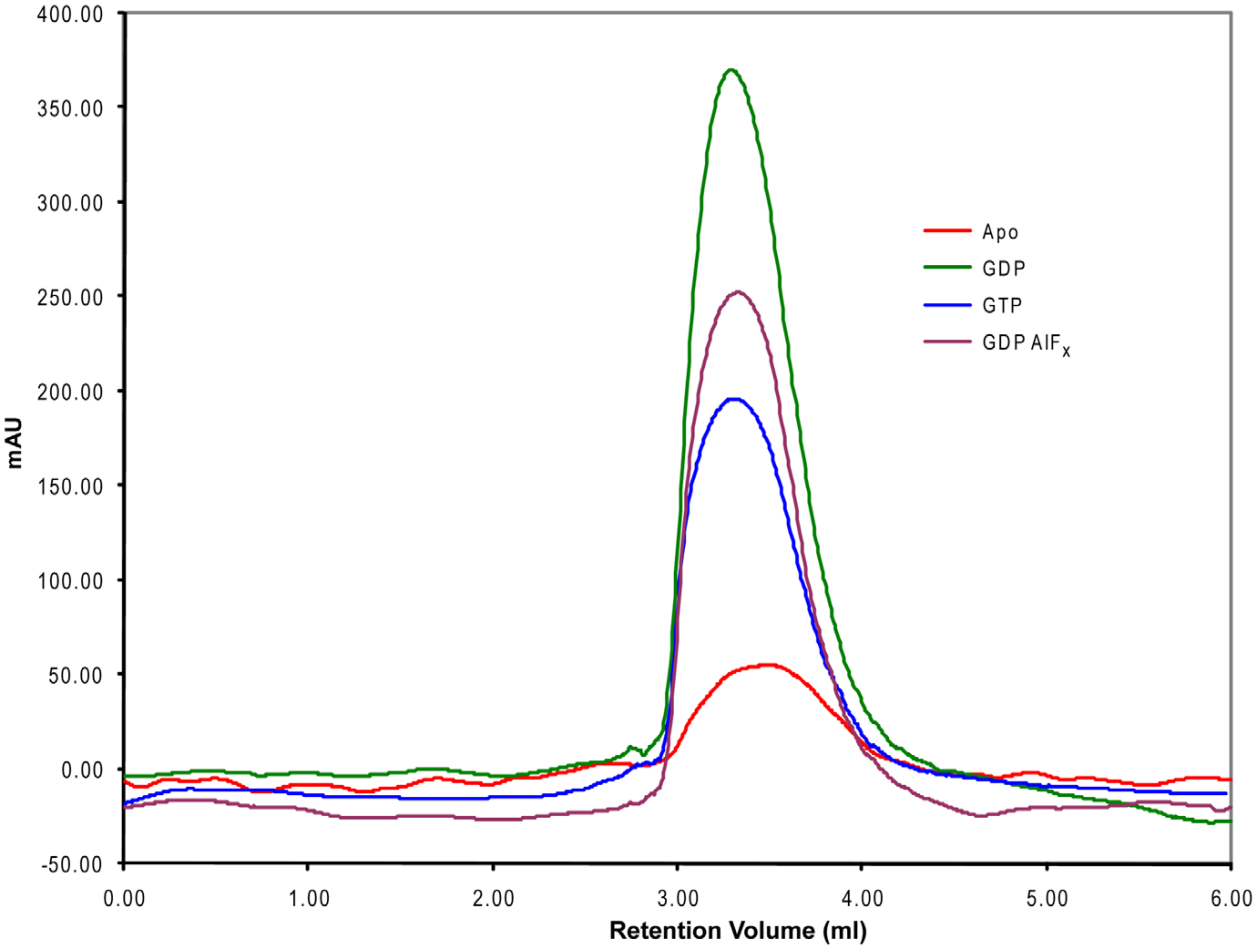
Probing oligomerisation of YqeH in the presence of nucleotides. Analytical gel filtration experiments were conducted in the apo (red curve) and in presence of GDP (green curve), GTP (blue curve) and GDP·AlF_x_ (pink curve) bound states of YqeH. Absorbance at 280 nm is shown in milli absorbance unit (mAU). The elution profile of YqeH in nucleotide-free apo state (red curve) indicates that it is a monomer. This profile remains unaltered in the different nucleotide bound states.

While it appears that K^+^ stabilizes the transition state, we additionally inquired whether YqeH too, like MnmE, oligomerizes in the presence of GDP·AlF_x_. Therefore, using full length YqeH, we tested this possibility in the nucleotide-free, GDP, GTP and GDP·AlF_x_ states. However, YqeH elutes as a monomer in all of these states (Fig. 3), which is in contrast to the transition state specific dimerization of the MnmE G-domain.

### Residues Important for Potassium Co-ordination

Encouraged by the finding that potassium stabilizes the transition state and influences GTP hydrolysis in YqeH, we set out to probe the residues critical for potassium co-ordination. The transition state structure of MnmE [23] showed that the hexa-coordinated potassium ion is held in the active site by interactions with K-loop (a part of the switch-I region), α and β phosphates of GDP, the side chain carbonyl oxygen of Asn-226 from P-loop and the fluoride ion from AlF_x_ (that mimics γ-phosphate). In order to determine, if such an arrangement is conserved in YqeH too, a combination of homology modeling and site-directed mutagenesis was employed.

**Figure 4:**
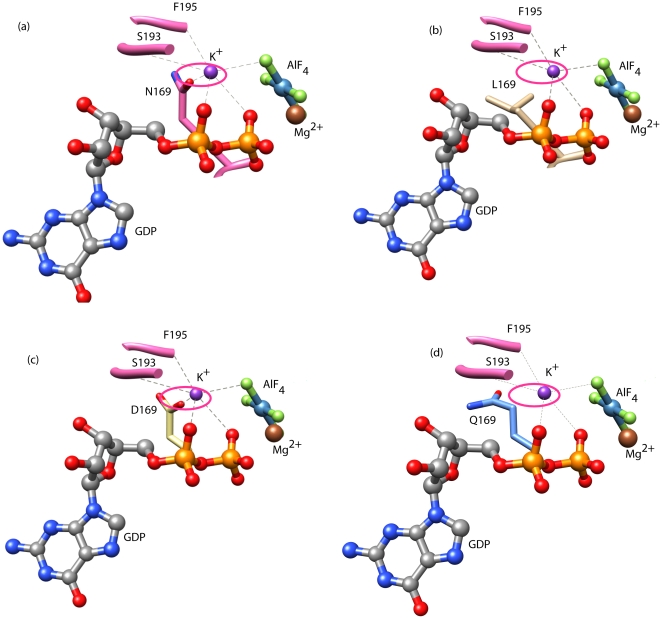
Hexa-coordinated potassium ion in the homology model of YqeH. GDP·AlF_x_ is shown in ball and stick model. The active site residues are displayed as sticks. Potassium is shown as a violet ball and its co-ordination with residues is indicated by dotted gray lines. Asn-169, one of the ligands coordinating potassium, is shown. Magnesium is shown as a brown ball. (a) YqeH model shows that potassium is held in place by coordination with α, β and γ phosphates of GTP, main chain interaction from the K-loop residues, S193 and F195, and side chain interaction with the amide group of Asn-169. The interaction with Asn-169 is highlighted by the pink circle. (b) N169L mutant that lacks the side chain carbonyl oxygen leads to loss of interaction with potassium. (c) N169D that retains the carbonyl oxygen and therefore the potassium coordination (d) N169Q, which despite possessing the carbonyl oxygen lacks potassium coordination due to an increased side chain length. Figures were made using chimera[37].

**Figure 5:**
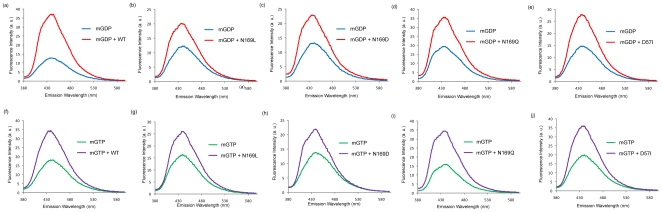
Nucleotide binding by YqeH Mutants. The ability of YqeH Mutants to bind nucleotides GTP and GDP is examined by measuring fluorescence intensity (in arbitrary units) of mant-GTP and mant-GDP (λ_ex_ - 355 nm, λ_em_ - 440 nm). The intrinsic and protein induced fluorescence of mGDP (blue and red curves, respectively) and mGTP (green and purple curves, respectively) is shown for (a & f) wild type, (b & g) N169L, (c & h) N169D, (d & i) N169Q and (e & j) D57I. Appreciable increase in fluorescence is observed for all mutants suggesting that they bind nucleotides.

A homology model for the CPG-domain of YqeH was generated using the transition state structure of the MnmE G-domain (monomer). This model suggested the likelihood that equivalent interactions stabilize the transition state in YqeH (Fig. 4a) and MnmE. To test this, Asn-169 (equivalent to Asn-226 in MnmE), one of the ligands coordinating the K^+^ ion through its side chain amide, was mutated to Leu, Asp and Gln, using site-directed mutagenesis. In order to assay their influence, if any, on nucleotide binding, fluorescent experiments were conducted using mGDP and mGTP. As shown in Figure 5, these mutants bind both mGDP and mGTP, suggesting that the mutations do not affect nucleotide binding (Fig. 5a–d, f–i). Based on the homology model, we reasoned that mutating Asn-169 to a hydrophobic Leu would disrupt its interaction with potassium and thereby would affect potassium dependent GTP hydrolysis (Fig. 4b). Indeed, the GTPase activity is completely abolished for the N169L mutant and could not be measured (Table 2). In contrast, we reasoned based on the model that mutating Asn-169 to Asp would preserve the co-ordination with potassium (Fig. 4c). Indeed, N169D retained GTPase activity comparable to wild type (Table 2). The effect of an increase in the side chain length was tested using N169Q mutation: Though Asn and Gln are chemically similar due to the presence of an amide group, the side chain is longer by 1.54 Å in Gln due to the presence of an additional CH_2_ group. Examining the model of YqeH, it appeared that this increase in side chain length would alter potassium co-ordination significantly (Fig. 4d). Indeed, the GTPase activity of N169Q mutant was completely abolished and could not be measured (Table 2). This finding concurs with a 72-fold reduction in activity seen upon mutating the corresponding Asn-226 to a Lysine in MnmE [23]. Overall, these studies underscore the importance of Asn-169 in coordinating the K^+^ ion.

**Table 2:** GTPase Activity of YqeH Mutants.

Construct	Specific Activity (min^−1^)
WT	1.1844±0.0227
N169L	**
N169D	0.9095±0.028
N169Q	**
D57I	**

Specific activity was measured in the presence of KCl and it is represented as the amount of Pi released for a given concentration of enzyme for a certain time (min). Value for wild type was taken from Table 1. Experiments were conducted in duplicates and were reproduced at least twice. The errors represent the standard deviation from the average. Values were corrected for the background intrinsic GTP hydrolysis. ** indicates that the activity could not be measured.

### Relocation of a Catalytic Residue due to the Circular Permutation

**Figure 6:**
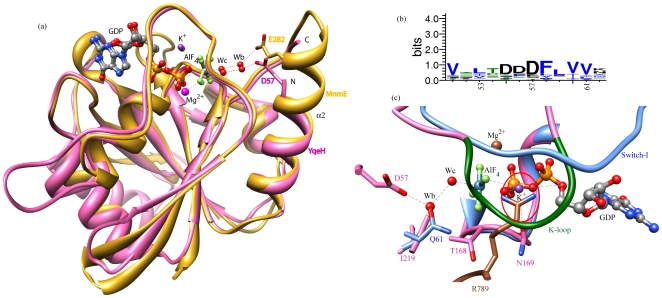
Relocation of the catalytic residue in YqeH. (a) YqeH homology model (pink) and MnmE (gold; PDB: 2gj8) are compared by substrate-based superposition. GDP·AlF_x_ is shown in ball and stick model. The active site residues are shown as sticks. The nucleophilic water (Wc) and bridging water (Wb) are indicated by red balls. Potassium (K^+^) is shown as a violet ball. Magnesium (Mg^2+^) is shown as a pink ball. Glu-282 in MnmE and Asp-57 in YqeH are indicated. The N and C-termini are shown for YqeH to appreciate the circular permutation. When compared to MnmE, Asp-57 from helix α2 is relocated to the N-terminus in YqeH due to the permutation. (b) The conservation of residues around Asp-57 at the N-terminal region of YqeH is displayed as a sequence logo [38] and the residue numbers are marked. The alignment of sequences from 26 orthologs that encompasses both bacteria and eukaryotes was made using MUSCLE [35] (c) The active site in YqeH homology model (pink) and Ras (blue; PDB: 1wq1) are compared by substrate-based superposition. Switch-I and K-loop (in green) are indicated. Arg-finger (Arg-789) from Ras-GAP is represented as stick model in brown. Magnesium (Mg^2+^) is shown as a pink ball. GDP·AlF_x_ is shown in ball and stick model. The active site residues are shown as sticks. The water mediated interaction from Asp-57 to catalytic water (Wc) is shown by a dotted gray line. The relative position of bridge water (Wb) coincides with that of the amide oxygen in Gln-61 of Ras. In contrast to Gln-61 in Ras, the corresponding position in YqeH is occupied by hydrophobic I219, which is retracted away from the catalytic site. The location of potassium overlaps with the position of Arg-finger (R789) in Ras-GAP and it is highlighted by a red circle. It may be seen that the presence of K-loop sterically occludes the approach of an Arg-finger like residue in YqeH.

While the mutational experiments suggest a likely equivalence in both YqeH and MnmE to stabilize the K^+^ ion, how the hydrolysis is achieved in YqeH remained unclear. The absence of catalytic glutamine (equivalent to Gln-61 in Ras) in YqeH indicates that it possesses an alternative means to achieve GTP hydrolysis. While in Ras, Gln-61 directly orients a catalytic water molecule to trigger GTP hydrolysis, in MnmE, Glu-282, (from helix α2) via an intermediate bridging water molecule, indirectly orients the analogous catalytic water [23]. However, a residue equivalent to Glu-282 is not found in YqeH. Adding to this, the region corresponding to helix α2 is relocated towards the N-terminus owing to the circular permutation (Fig. 1 & 6a), making the identification of the catalytic residue a non-trivial exercise based on sequence information alone. However, a careful examination for conserved residues in the N-terminal region of YqeH together with the recently determined structures of YqeH orthologs GsYqeH from *Geobacillus sterothermophilus* [24] and BaYqeH from *Bacillus anthracis* (Brunzelle et al., unpublished; PDB: 3h2y), led us to reason Asp-57 to be a potential catalytic residue (Fig. 6a & 6b; see discussion). A homology model was made considering Asp-57 to be an equivalent of Glu-282 in MnmE (Fig. 6a). From this model, it was possible to envisage a water mediated interaction facilitating GTP hydrolysis in YqeH, similar to that in MnmE (Fig. 6a & 6c). To test this possibility, a point mutant D57I was created and assayed for its ability to hydrolyse GTP. Though D57I mutant retained mGDP and mGTP binding (Fig. 5e & j), GTP hydrolysis was completely abolished (Table 2), strengthening the view that Asp-57 may indeed facilitate catalysis in YqeH.

## Discussion

Structural and biochemical studies on Ras-like GTPases showed that participation of both (i) catalytic glutamine (*in cis*) as well as the so called (ii) Arg-finger (*in trans*) are two key aspects driving GTP hydrolysis [2], [22], [25], [26]. An importance for these was reinforced by mechanistic studies on several other GTPases, like G-α subunit of heterotrimeric G-proteins, Rho, Rab33, Ran and Arf [27], [28], [29], [30], [31], although variations were noted. However, the absence of a catalytic glutamine in HAS-GTPases like YqeH, and the absence of any known GAP(s), suggests that it is likely to employ an alternative means to achieve catalysis. In line with this, we find that like in MnmE, potassium activates GTPase activity in YqeH too (Table 1). This prompted us to utilize the information provided by the transition state structure of MnmE to understand the structural basis for the role of potassium in GTP hydrolysis. The homology model of YqeH when overlaid onto the transition state structure of Ras[22] (PDB: 1wq1) showed that the location of potassium overlaps with the position of Arg-finger in Ras (Fig. 6c). Like in MnmE [23], this raises the possibility that in YqeH too, potassium could play a role akin to Arg-finger of Ras to stabilize the transition state. Indeed, fluorescent binding experiments conducted in the presence of mGDP·AlF_x_ (a transition state mimic) and different salts (Na^+^, K^+^, NH4^+^, Rb^+^, Cs^+^) suggests that K^+^, NH4^+^ and Rb^+^ stabilize the transition state (Fig. 2) and thereby accelerate GTPase activity (Table 1). The order in which these ions influence GTP hydrolysis is as follows: K^+^ > NH4^+^ > Rb^+^ > Cs^+^ > Na^+^. This suggests that ionic radius of sodium (102 pm), which is smaller than that of potassium (138 pm) cannot promote GTP hydrolysis (Table 1), depicting a clear specificity for potassium. On the other hand, cesium with a radius 167 pm displays a 10 fold lower activity than K^+^. By analyzing the transition state structure of MnmE, Scrima and Witttinghofer [23] suggested that the active site may utilize monovalent cations with radii between 138-152pm. That NH4^+^ (147 pm) and Rb^+^ (152 pm) also elicit similar effects as K^+^ on GTP hydrolysis, may further indicate analogous K^+^ binding in YqeH and MnmE. Conversely, an absence or sub-optimal GTPase activity with sodium and cesium may be because the ionic radii of these ions do not fall within the aforesaid limit (Table 1). While it seems that active site dimension contributes to potassium specificity, inspection of the active site of YqeH model suggests that like in MnmE, potassium is bound at the active site by main chain interactions with a part of switch-I, termed the K-loop, α and β phosphates of GDP, a fluoride ion (*i.e.* the γ-phosphate mimic) and the side chain of Asn-169 from P-loop (Fig. 4a). Mutational studies on Asn-169 render support to the modeling studies that indeed Asn-169 in YqeH contributes to potassium coordination (Fig. 4 & 5; Table 2).

While a small ion like potassium participates in GTP hydrolysis, it is not clear whether an Arg-finger like mechanism is also possible in YqeH. Analysis of the modeled structure indicates that the presence of K-loop would sterically occlude the entry of an Arg side chain of a GAP (Fig. 6c). However, it is important to note that the homology model derived for YqeH rests on the assumption that the K-loop adopts a conformation similar to MnmE. The aforesaid mutational studies of Asn-169, allow us to believe that this assumption is perhaps reasonable. Therefore, it appears unlikely that an Arg-finger mediated mechanism operates in presence of a K-loop that adopts a conformation to shield its entry. As a result, it seems more appropriate to have a small ion like potassium to stimulate the otherwise weak intrinsic GTP hydrolysis.

Furthermore, in MnmE, catalysis is facilitated by the dimerization of its G-domain [23]. This led us to investigate whether full length YqeH also dimerizes in a K^+^ dependent manner to hydrolyze GTP. Analytical gel filtration experiments conducted in the presence of GDP, GTP and GDP·AlF_x_ showed no alteration in elution profile suggesting that YqeH remains a monomer in the presence of K^+^ and GDP·AlF_x_ (Fig. 3). This presents the first mechanistic difference between YqeH and MnmE. Although both employ a K^+^ dependent catalysis, YqeH is likely to achieve it without dimerization (see below).

Apart from potassium, another important component of GTP hydrolysis is the catalytic residue. The lack of a catalytic Gln at a position equivalent to Gln-61 in Ras, prompted us to identify a likely candidate in YqeH. Thus together with sequence and structural analysis of YqeH, homology modeling was initiated using the monomeric transition state structure of MnmE as the template (Fig. S1, S2, S3; Fig 6a). Superposition of the G-domains of MnmE (transition state) and YqeH (GDP-bound) showed that while switch-II in MnmE is continuous with helix α2, in YqeH, owing to the circular permutation, it is relocated towards the C-terminus and is connected to the C-terminal PNR domain (Fig. S2 & S3). For the same reason, helix α2 in YqeH is also relocated towards the N-terminus where it is connected to a Zn finger domain (Fig. S3). Since the conformation of switch-II differs between YqeH and MnmE, accurate modeling of switch-II in YqeH, based on MnmE, is not possible (Fig. S2). However, the structural superposition revealed that, in YqeH, the residue equivalent to the catalytic Glu-282 of MnmE, may not satisfy an analogous role due to a steric clash with Ile-220 (Fig. S2). Therefore, unlike in MnmE, the catalytic residue may not be presented from the first turn of helix α2. Instead, three highly conserved Asp residues, Asp-55, Asp-56 and Asp-57 were chosen to be likely candidates. Of these, due to the periodicity of the α-helix (residue i and residue i+4 will be located on same side of the α-helix), the only other residue that could stabilize the bridging water appeared to be Asp-57 from helix α2 (which corresponds to Gly-285 in MnmE). Apart from this, we note a possibility that Asp-55 and Asp-56 may interact with Asn-221, Asn-222 and His-223 of Switch-II, whose chemical nature is strongly conserved. Like the interaction between Arg-275 and Glu-282 in MnmE, this interaction in YqeH may help to orient helix α2 suitably and thus position Asp-57 competent to coordinate the bridge water (Fig. S1 & S3). Although speculative, this perhaps explains the conservation of Asp-55 and Asp-56 along with Asp-57 (Fig. 6b). However, a detailed structural investigation in the transition state would be required to understand the underlying mechanism. Nevertheless, the role of Asp-57 in catalysis is substantiated by the finding that D57I mutation allows nucleotide binding, but abolishes all catalytic activity (Fig. 5e & j; Table 2). In summary, it appears that Asp-57 in YqeH facilitates water mediated catalysis similarly to the Glu-282 in MnmE, except that owing to the circular permutation, it is presented from the N-terminal region (Fig. 6a) and therefore its interactions with switch-II are expected to be different. Despite this variation in topologies between MnmE and YqeH, the catalytic residues Glu/Asp are presented from similar regions (helix α2), reflecting a structural plasticity necessary to preserve GTP hydrolysis.

Scrima and Wittinghofer[23] observe Era and EngA to possess a K-loop like feature including the conserved “GTTRD” motif and a conserved Asn in the P-loop (corresponding to Asn-169 in YqeH): We observe these features to be present in the ribosome binding cpGTPases YlqF and YawG too. In MnmE, this motif provides important interactions to mediate catalysis. Gly-249 is highly conserved and possesses torsion angles that are disallowed for non-glycyl residues[23]. It coordinates the γ-phosphate and also orients the catalytic water. Thr-250 binds γ-phosphate through main chain interactions and Thr-251 (corresponding to Thr-35 in Ras) coordinates the γ-phosphate and also the Mg^2+^. Given this importance for the “GTTRD” motif [32], its conservation in cpGTPases YlqF and YawG that also participate in ribosome assembly, tempts us to speculate that they too would utilize a water mediated interaction coupled with a small monovalent cation like K^+^ to drive GTP hydrolysis.

This work allows us to conclude that GTP hydrolysis in HAS-GTPases like YqeH and MnmE is driven by water mediated interactions facilitated by Asp/Glu (*in cis*) as well as the participation of a small monovalent potassium ion (*in cis*). This is in contrast to the participation of Gln (*in cis*) and Arg-finger (*in trans*) in Ras-like GTPases. These variations indicate that there is more than one way to achieve GTP hydrolysis and reveal a new class of GTPases that utilize an alternative catalytic mechanism than that seen for well studied GTPases like Ras.

## Materials and Methods

### Site Directed Mutagenesis

Site-directed mutagenesis was performed using a megaprimer approach[33]. The mutant PCR product was generated by using the appropriate mutant primers along with the full length (residues 1–366) primers (Table S1) using Pfu DNA polymerase (fermentas). Cloning into the modified pGEX4T1 vector (GE Healthcare) was performed using restriction sites *Nhe1* and *Xho1* (NEB). Mutations were confirmed by DNA sequencing and using restriction digestion specific for a particular mutation if such a recognition site is available. Expression and purification of the GST fusion constructs were performed as described previously[19]. As reported previously[19], since GST does not affect nucleotide and ribosome binding, all assays for the mutants were also conducted using GST fusion constructs.

### Nucleotide Binding and GTPase Assays

The nucleotide binding assay was performed using fluorescent mant nucleotides as described[19]. The reaction mixture (150 µl) consisted of 20 mM Tris pH 8, 5 mM MgCl_2_, 100 mM salt (NaCl/KCl/NH_4_Cl/RbCl/CsCl), 500 nM mGDP/mGTP and 1 µM YqeH. For transition state studies, 5 µM NaF and 500 nM AlCl_3_ were also included in the reaction mixture.

GTPase assays were performed using the malachite green method as described[19]. The reaction mixture (50 µl) consisted of 400 nM protein and 400 µM GTP in 20 mM Tris pH 8, 100 mM Salt (NaCl, KCl, NH_4_Cl, RbCl and CsCl), 5 mM MgCl_2_ and 5 mM 2-mercaptoethanol.

### Analytical Gel Filtration

To test the oligomerisation using analytical gel filtration, YqeH was cloned into pQE2 vector (Qiagen) using *NdeI* and *HindIII* restriction sites to generate a His-tagged fusion protein. The protein was over-expressed in *E.coli* DH5α cells and purification was done using Ni-sepharose column (Amersham Biosciences) by essentially following the protocol as described previously[19] for GST fusion constructs, except that the protein was eluted using a linear gradient of imidazole (20 mM–500 mM) in 50 mM Tris-Cl pH 8, 150 mM NaCl, 5 mM 2-mercaptoethanol. The protein was further purified using Superdex 200 Highload 26/60 column (Amersham Biosciences). The column was pre-equilibrated with 50 mM Tris-Cl pH 8, 150 mM KCl, 5 mM 2-mercaptoethanol and protein was eluted in the same buffer and was concentrated using Amicon ultrafiltration columns (Millipore). During this process, the buffer was slowly exchanged with 50 mM Tris-Cl pH 8, 150 mM NaCl, 5 mM 2-mercaptoethanol, aliquoted and stored at −80°C until required. Protein concentration was estimated using BCA assay (Sigma).

Analytical gel filtration experiments were performed using 7 µM YqeH in 20 mM Tris pH 8, 10 mM MgCl_2_ and 150 mM KCl for the nucleotide-free state. For the nucleotide bound states, 1 mM GDP/GTP and for the transition state analysis 1 mM GDP with 1 mM AlCl_3_ and 10 mM NaF were included, in the above mixture. The reaction mixture (100 µl) was incubated at room temperature for 10 min and loaded onto a Superdex 200 5/150 GL column (Amersham Biosciences).

### Homology Modeling

Homology modeling was performed using modeller9v2 [34]. The monomeric form of MnmE G-domain (PDB: 2gj8), determined in the transition state, was used as a template. Each chain (residues 217–376) corresponding to the G-domain was used as a separate template. Since MnmE is not circularly permuted like YqeH, the G-domain of MnmE was artificially permuted for aligning the sequence with the corresponding CPG-domain of YqeH (residues 57–222). Consequently, switch-II region in YqeH is not accurately modeled. The alignment was made using MUSCLE [35] and was manually adjusted later. Distance restraints between active site residues and GDP·AlF_x_ were derived from MnmE structure and were applied to the equivalent residues in YqeH. Based on the evaluation of stereo chemical violations using MolProbity [36] as well as using Modeller's discrete optimized protein energy (DOPE) potential, the best model was chosen for further analysis. Approximately 94% of the residues in the chosen models fall under the favoured region in Ramachandran plot. RMSD for the YqeH homology model and the MnmE transition state structure based on 88 equivalent atom pairs is 0.88 Å.

## Supporting Information

Figure S1 *Comparison of helix α 2 in the nucleotide-free and transition state structures of the MnmE G-domain*. MnmE in apo (PDB: 1xzp) and GDP.AlF_x_ (PDB: 2gj8) bound transition state are shown in pink and blue ribbons, respectively. Amino acid side chains are shown as sticks. Mg^2+^ (pink ball), K^+^ (purple ball), AlF_x_ (green ball and stick), GDP (ball and stick with P in orange, O in red and C in gray), catalytic water (red ball, indicated by cw) and bridge water (red ball, indicated by bw) are shown. The position of helix α 2 is indicated for both apo and transition state structures. This superposition reveals a rearrangement at the N-terminal region of helix α 2 to position the catalytic residue, E282, in a manner competent to stabilize the bridge water (see the position of E282 in apo and GDP.AlF_x_ bound states). The orientation of E282 is further stabilized by interaction with R275 of Switch-II, which also depicts a large change in its position between the two states.
(3.34 MB TIF)

Figure S2 *Conformation of switch-II in MnmE (transition state) and GDP bound YqeH*. MnmE in GDP•AlF_x_ bound state (PDB: 2gj8) and *Bacillus anthracis* YqeH (BaYqeH) in dGDP bound state (PDB: 3h2y) are shown in blue and brown ribbons, respectively. Representation of amino acids and other elements follows Figure S1. The positions of helix α 2 and switch-II (sw-II) are indicated for MnmE, while only the Switch-II is shown for YqeH due to a relocation of helix α 2 owing to the circular permutation (see Fig. S3). The location of C-terminal PNR domain in YqeH is depicted by a purple square. *Bacillus subtilis* YqeH residue numbering is used throughout. The steric clash between E282 in MnmE and I220 in YqeH is depicted by a pink sphere. In MnmE, the switch-II is continuous with helix α 2. However, owing to circular permutation in YqeH, switch-II is relocated towards the C-terminus and it is now connected to PNR domain. Therefore, the conformation of switch-II is different in MnmE and YqeH. As a consequence, E282 like residue cannot be presented from the first turn of helix α 2 to bridge the intermediate water due to a steric clash with I220. The alternative location to present the potential catalytic residue from helix α 2 would be residue 286 (since i and i+4th residues are on the same side of the α-helix). However, position corresponding to 286 in MnmE (Ile) and YqeH (Phe) is hydrophobic in nature. Hence, the likely residue corresponds to position 285, located in second turn of the helix in MnmE, which is a glycine. This requires a reorientation of helix α 2, as suggested by Figure S3. This position in YqeH corresponds to Asp57, the proposed catalytic residue.
(2.39 MB TIF)

Figure S3 *A hypothesis concerning the re-orientation and stabilization of helix α 2 in YqeH*. MnmE in GDP•AlF_x_ bound state (PDB: 2gj8) and *Bacillus anthracis* YqeH (BaYqeH) in dGDP bound state (PDB: 3h2y) are shown in blue and light pink ribbons, respectively. Representation of amino acids and other elements follow Figure 1. The positions of helix α 2 and switch-II (sw-II) are indicated. The location of C-terminal PNR domain in YqeH is depicted by a purple square and that of N-terminal Zn finger domain by a brown square. *Bacillus subtilis* YqeH residue numbering is used throughout. Comparison of helix α 2 in MnmE and YqeH suggests that D55 of YqeH corresponds to R283 of MnmE, D56 of YqeH corresponds to I284 of MnmE and D57 of YqeH corresponds to G285 of MnmE. Inspection of electron densities for D55, D56 and D57 using the deposited structure factors (PDB: 3h2y) shows that the electron density for the side chains of these residues is not well resolved. This indicates that these residues are mobile. However, based on the periodicity of α-helix (i^th^ residue and i+4^th^ residue occupies the same face of the helix) and maping the location of D55, D56 and D57 onto helix α 2 of MnmE, it is possible to suggest that D55 and D56 are unlikely to bridge the intermediate water as they would be oriented away from the catalytic pocket. In that case, it is intriguing how helix α 2 and D57 could be reoriented. Inspection of switch-II in YqeH shows that N221, H222 and H223 could interact with D55, which might help orienting helix α 2 suitably. This possibility gains strength from the fact that, like D55, D56 and D57, the chemical nature of positions 221–223 is also conserved across YqeH orthologs. Since helix α 2 is connected to Zn-finger domain in YqeH, a possible domain movement associated with it could also help reorienting helix α 2 and thereby position D57 in a catalytically competent position.
(2.43 MB TIF)

Table S1 List of primers used in generating YqeH point mutants.
(0.03 MB DOC)
