# Supplementary figures and images for: Deciphering the Catalytic Machinery in 30S Ribosome Assembly GTPase YqeH
Source: PLoS One. 2010 Apr 1;5(4):e9944. doi: 10.1371/journal.pone.0009944 (PMC2848588; doi:10.1371/journal.pone.0009944)

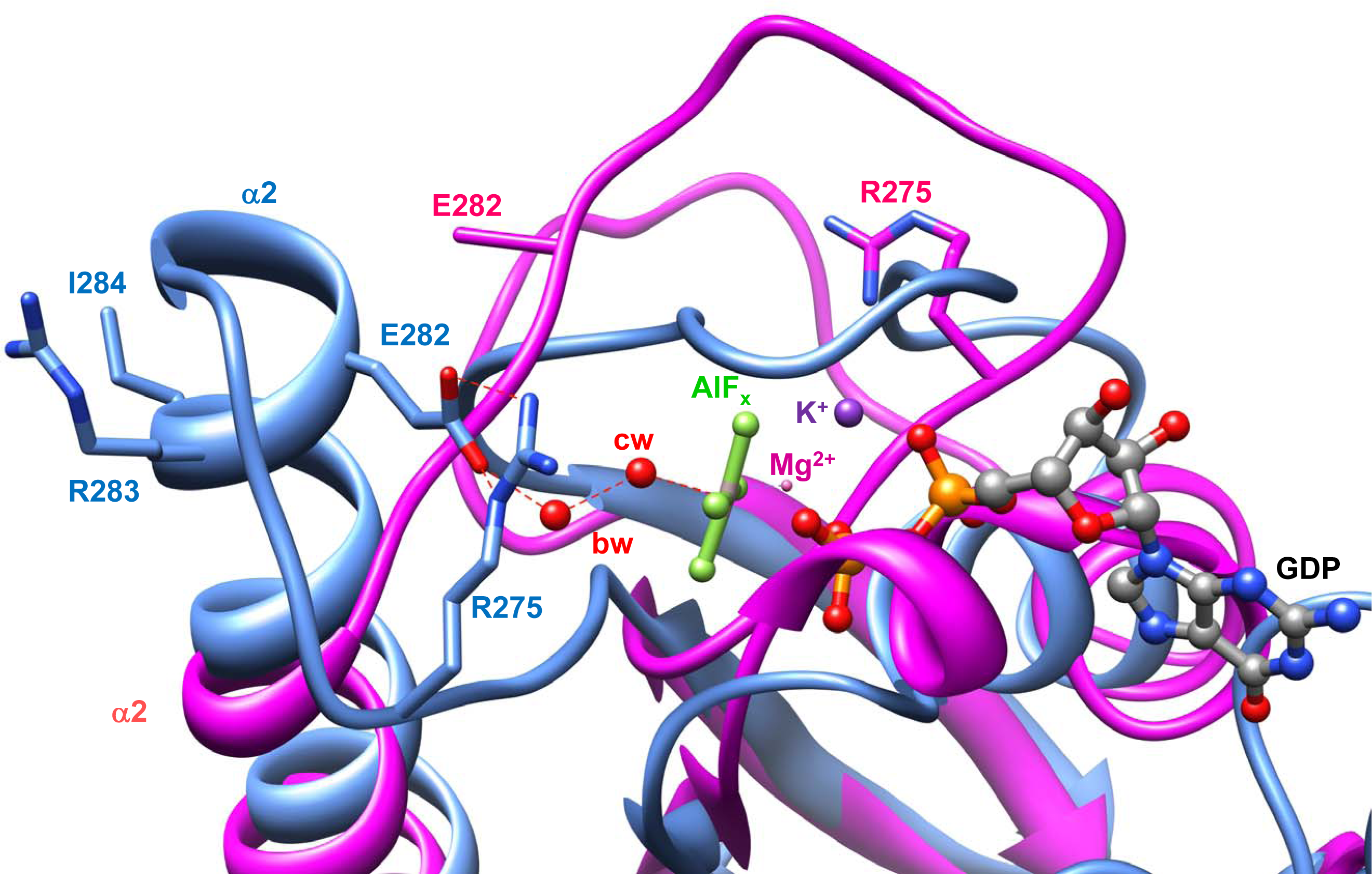

Supplement: Figure S1 — Comparison of helix α 2 in the nucleotide-free and transition state structures of the MnmE G-domain. MnmE in apo (PDB: 1xzp) and GDP.AlFx (PDB: 2gj8) bound transition state are shown in pink and blue ribbons, respectively. Amino acid side chains are shown as sticks. Mg2+ (pink ball), K+ (purple ball), AlFx (green ball and stick), GDP (ball and stick with P in orange, O in red and C in gray), catalytic water (red ball, indicated by cw) and bridge water (red ball, indicated by bw) are shown. The position of helix α 2 is indicated for both apo and transition state structures. This superposition reveals a rearrangement at the N-terminal region of helix α 2 to position the catalytic residue, E282, in a manner competent to stabilize the bridge water (see the position of E282 in apo and GDP.AlFx bound states). The orientation of E282 is further stabilized by interaction with R275 of Switch-II, which also depicts a large change in its position between the two states. (3.34 MB TIF) [file pone.0009944.s001.tif]

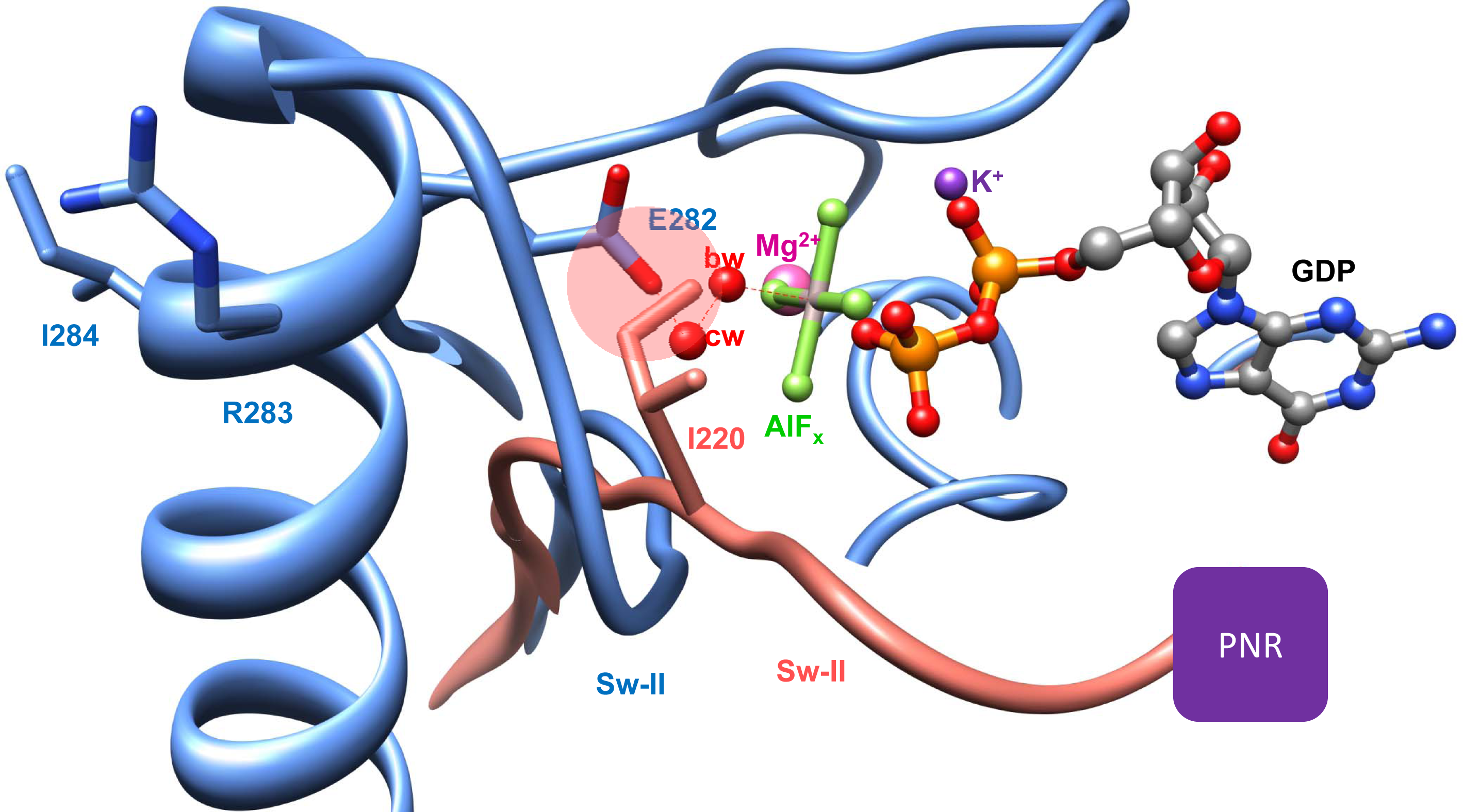

Supplement: Figure S2 — Conformation of switch-II in MnmE (transition state) and GDP bound YqeH. MnmE in GDP•AlFx bound state (PDB: 2gj8) and Bacillus anthracis YqeH (BaYqeH) in dGDP bound state (PDB: 3h2y) are shown in blue and brown ribbons, respectively. Representation of amino acids and other elements follows Figure S1. The positions of helix α 2 and switch-II (sw-II) are indicated for MnmE, while only the Switch-II is shown for YqeH due to a relocation of helix α 2 owing to the circular permutation (see Fig. S3). The location of C-terminal PNR domain in YqeH is depicted by a purple square. Bacillus subtilis YqeH residue numbering is used throughout. The steric clash between E282 in MnmE and I220 in YqeH is depicted by a pink sphere. In MnmE, the switch-II is continuous with helix α 2. However, owing to circular permutation in YqeH, switch-II is relocated towards the C-terminus and it is now connected to PNR domain. Therefore, the conformation of switch-II is different in MnmE and YqeH. As a consequence, E282 like residue cannot be presented from the first turn of helix α 2 to bridge the intermediate water due to a steric clash with I220. The alternative location to present the potential catalytic residue from helix α 2 would be residue 286 (since i and i+4th residues are on the same side of the α-helix). However, position corresponding to 286 in MnmE (Ile) and YqeH (Phe) is hydrophobic in nature. Hence, the likely residue corresponds to position 285, located in second turn of the helix in MnmE, which is a glycine. This requires a reorientation of helix α 2, as suggested by Figure S3. This position in YqeH corresponds to Asp57, the proposed catalytic residue. (2.39 MB TIF) [file pone.0009944.s002.tif]

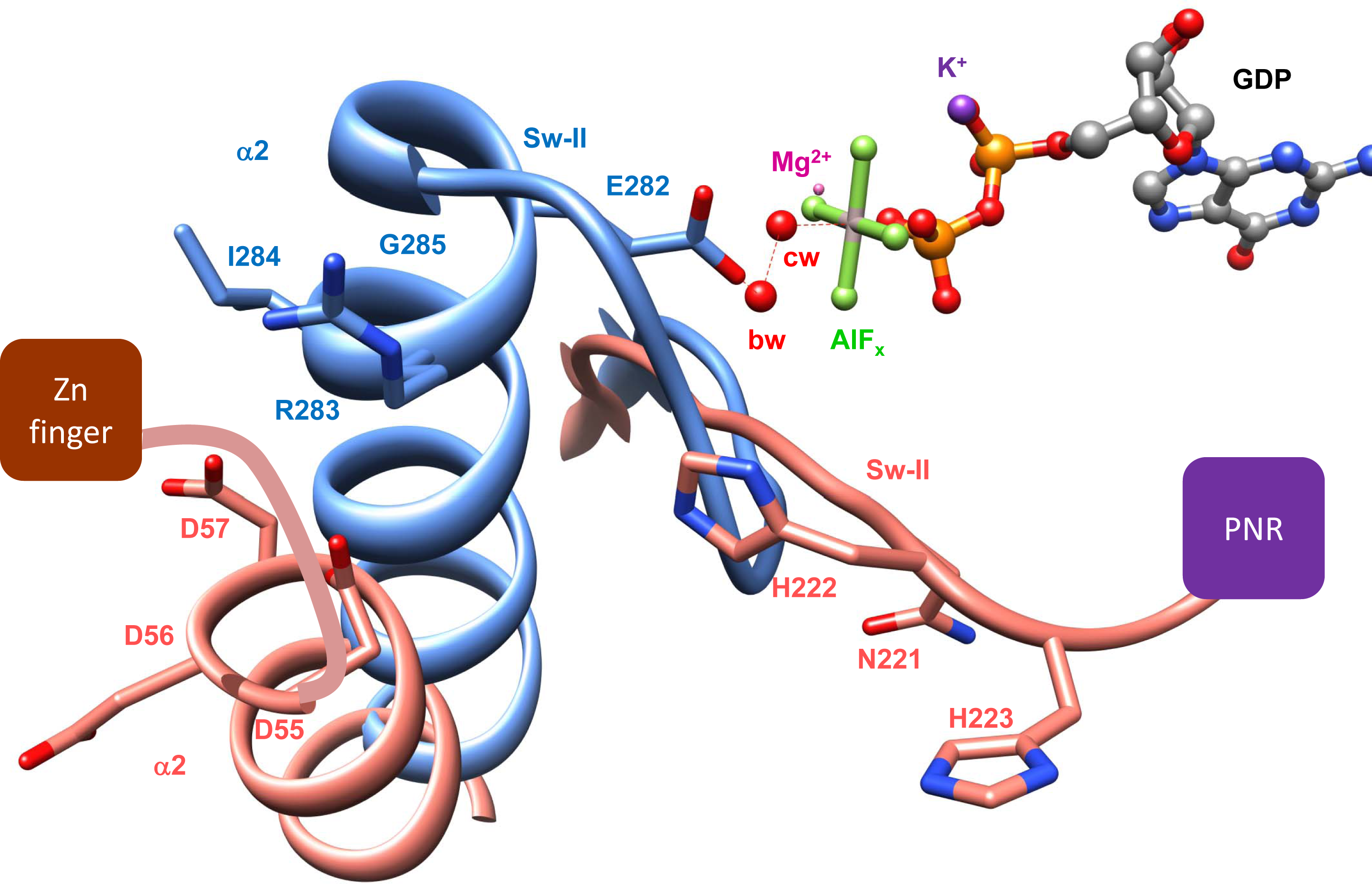

Supplement: Figure S3 — A hypothesis concerning the re-orientation and stabilization of helix α 2 in YqeH. MnmE in GDP•AlFx bound state (PDB: 2gj8) and Bacillus anthracis YqeH (BaYqeH) in dGDP bound state (PDB: 3h2y) are shown in blue and light pink ribbons, respectively. Representation of amino acids and other elements follow Figure 1. The positions of helix α 2 and switch-II (sw-II) are indicated. The location of C-terminal PNR domain in YqeH is depicted by a purple square and that of N-terminal Zn finger domain by a brown square. Bacillus subtilis YqeH residue numbering is used throughout. Comparison of helix α 2 in MnmE and YqeH suggests that D55 of YqeH corresponds to R283 of MnmE, D56 of YqeH corresponds to I284 of MnmE and D57 of YqeH corresponds to G285 of MnmE. Inspection of electron densities for D55, D56 and D57 using the deposited structure factors (PDB: 3h2y) shows that the electron density for the side chains of these residues is not well resolved. This indicates that these residues are mobile. However, based on the periodicity of α-helix (ith residue and i+4th residue occupies the same face of the helix) and maping the location of D55, D56 and D57 onto helix α 2 of MnmE, it is possible to suggest that D55 and D56 are unlikely to bridge the intermediate water as they would be oriented away from the catalytic pocket. In that case, it is intriguing how helix α 2 and D57 could be reoriented. Inspection of switch-II in YqeH shows that N221, H222 and H223 could interact with D55, which might help orienting helix α 2 suitably. This possibility gains strength from the fact that, like D55, D56 and D57, the chemical nature of positions 221–223 is also conserved across YqeH orthologs. Since helix α 2 is connected to Zn-finger domain in YqeH, a possible domain movement associated with it could also help reorienting helix α 2 and thereby position D57 in a catalytically competent position. (2.43 MB TIF) [file pone.0009944.s003.tif]
