# Supplementary material for: Deciphering the Catalytic Machinery in 30S Ribosome Assembly GTPase YqeH
Source: PLoS One. 2010 Apr 1;5(4):e9944. doi: 10.1371/journal.pone.0009944 (PMC2848588; doi:10.1371/journal.pone.0009944)
Supplement: Table S1 — List of primers used in generating YqeH point mutants. (0.03 MB DOC) [file pone.0009944.s004.doc]

**Table S1**

**List of primers used in generating YqeH point mutants**

| **Constructs** | **Primers used*** |
| --- | --- |
| N169L | RP: 5’-AGACGTCTATGTTGTCGGATGTACA**CTA**GTAGGAAAGTCAACCTTTATTAACC-3' |
| N169D | RP: 5'- GTTGTCGGATGTAC**CGAT**GTAGGAAAGTC-3' |
| N169Q | RP: 5’-GTTGTCGGATGTACA**CAG**GTAGGAAAGTCAA-3’ |
| D57I | RP: 5’-GTCTCCTTAACTGATGAT**ATC**TTTTTGAACATTCTTCACGGT-3’ |
| FP | 5’- CTA*GCTAGC* TGGAAAAGGTTGTTTG-3’ |
| RP | 5’-CCG*CTCGAG* AAATTAAGGAACGCCGAAC-3’ |

*The region containing the mutations is indicated in bold.

FP – forward primer; RP – Reverse primer

The recognition sites for *NheI* (GCTAGC) and *XhoI* (CTCGAG), are indicated in italics.

For cloning into pQE2, *NdeI* and *HindIII* were used.
